# Subjective Performance Expectations From and Demographic and Categorical Differences in the Acceptance of Virtual Reality or AI Technologies in Rehabilitation Programs: Cross-Sectional Questionnaire Survey With Rehabilitation Patients

**DOI:** 10.2196/69350

**Published:** 2025-08-08

**Authors:** Guido Waldmann, Dominik Raab

**Affiliations:** 1 MEDICLIN Rehabilitation Center, MEDICLIN Reha Research, Gustav-Adolf-Straße 15, Bad Düben, 04849, Germany, 49 34243792212, 49 342432216; 2 Chair for Mechanics and Robotics, University of Duisburg-Essen, Duisburg, Germany

**Keywords:** virtual reality, expectation of success, computer-assisted rehabilitation, motivation, neurologic rehabilitation

## Abstract

**Background:**

More than a few concepts have been presented in rehabilitation clinics that implement aspects of modern IT in the arrangement of augmented reality or virtual rehabilitation aiming to enhance cognitive or motor learning and rehabilitation motivation. Despite their scientific success, it is currently unknown whether rehabilitants will accept rehabilitation concepts that integrate modern ITs.

**Objective:**

This study aims to investigate the subjective performance expectations of rehabilitation patients regarding the application of virtual reality (VR) or artificial intelligence technologies across various therapeutic fields, and to identify demographic and categorical differences in acceptance to inform the development and implementation of VR-based rehabilitation programs.

**Methods:**

In total, 111 rehabilitation patients were surveyed about their subjective performance expectations of VR in 15 therapeutic fields with a questionnaire. The distribution of the responses was evaluated using box plots. The relationship between the subjective performance expectations for the 15 therapeutic fields was analyzed using the Spearman ρ coefficient, while the Mann-Whitney *U* test was used to compare subjective performance expectations between age groups and between genders.

**Results:**

For all 15 therapeutic fields, the median of the subjective performance expectations was between 2 and 3, while therapeutic fields in the categories “activity/movement,” “competence in daily life/communication,” and “education” tended to be rated higher than therapeutic fields in the categories “relaxation/passive measures” and “advisory/conversation.” A significant rank correlation was observed for 103 out of 105 pairwise comparisons of the therapeutic fields, with distinct patterns of effects sizes within the chosen categories. There was no significant difference in the evaluation between rehabilitants of employable age and those aged 68 years or older. Male rehabilitation patients reported greater subjective expectations for virtual rehabilitation than female patients, but there was only a significant difference with small effect sizes for 3 of the 15 therapeutic fields.

**Conclusions:**

The general trend is that patients can imagine taking part in VR in rehabilitation activities involving active movement (physiotherapy, sports and exercise therapy, and occupational therapy) and health education. The results of the survey show that there is also a high level of support for the therapeutic field advisory/conversation. Current circumstances have led to substantial use of virtual offerings in practice. The limited data available may have encouraged the professional development of VR systems and their widespread use in medical rehabilitation follow-up in the home setting.

## Introduction

Didactic principles and learning strategies play a vital role in medical rehabilitation, whereby the path for knowledge acquisition differs for young and old individuals. These age-specific differences in learning appear to be due to changes in neural systems that assess how much should be learned from changes in the environment [1]. In rehabilitation, the advantages of virtual environments and training programs with a high interactive content can be used for such knowledge acquisition. Positive transfer effects from a virtual space to the real environment are already known. In addition, a virtual training environment can be designed to provide motivational feedback and to directly control the complexity of a therapeutic content or environmental changes according to the patient’s performance. In virtual environments, both lessons and practice sequences can be repeated as often as required, and incorrect actions are reversible and have no consequences. Learning from faults and training at performance limits can be easily controlled. Such learning environments are free of charge and, in principle, have unlimited availability.

Perception and learning under virtual reality (VR) conditions have been studied not only in healthy individuals but also in those with brain injury [2-6]. This indicates that approaches to VR in brain injury rehabilitation are already established in terms of cognitive assessment and cognitive rehabilitation with technically simple systems. However, VR systems have not yet been established in motor rehabilitation. Studies with immersive systems have shown at least preliminary positive effects in upper extremity rehabilitation training [6]. For a general integration into rehabilitation concepts, a comfortable technical applicability and obvious motion detection are central requirements. Therapist-assisted rehabilitation interventions are often superior to purely technical interventions or virtual environments in everyday life. The development of new motion recognition systems for the gaming industry in recent years has made it possible to develop new everyday conditions for the use of virtual environments in motor rehabilitation [7,8]. However, the motivation and acceptance of people confronted with this technology in a therapeutic setting have not been sufficiently investigated. Key questions in this context are as follows:

1. Can neurological rehabilitation patients imagine that virtual rehabilitation will be developed as a new rehabilitation concept?
2. How do rehabilitation patients rate this vision in terms of different therapeutic areas and perceived therapeutic success?
3. Can virtual training lead to increased motivation and cooperation among rehabilitation patients in terms of therapeutic participation or self-regulated training?

In complex models describing the probability of user acceptance with an innovation, subjective performance expectancy regarding the benefits of a system is the strongest predictor of behavioral intention to accept an innovation [9-11]. Therefore, in this work, a survey of the subjective performance expectancy of virtual rehabilitation is conducted to provide a basis for the systematic evaluation of the benefits that VR can bring to various areas of medical rehabilitation.

## Methods

### Research Questionnaire and Survey

A questionnaire was developed to measure patients’ subjective performance expectations from virtual rehabilitation in 15 therapeutic fields using a Likert scale [12] with ratings 1=excellent, 2=good, 3=adequate, 4=unsatisfactory, and 5=poor. Table 1 provides an overview of the selected therapeutic fields as well as a classification into 5 basic categories, illustrating that the full spectrum of therapeutic measures is covered by the questionnaire. In version 1.1 of the questionnaire, 2 questions were added: “Do you think that virtual reality can be used to achieve higher motivation for cooperation and training?” “Do you have any experience with computers or game consoles? (Yes/No)?”

**Table 1.** Categorization of therapeutic fields.

Therapeutic fields	Categories
Physiotherapy	Activity/movement
Sports and exercise therapy	Activity/movement
Occupational therapy	Competence in daily life/communication
Speech therapy	Competence in daily life/communication
Relaxation techniques	Relaxation/passive measures
Physical therapy	Relaxation/passive measures
Psychological individual therapy	Advisory/conversation
Psychological group therapy	Advisory/conversation
Discussion groups on disease management	Advisory/conversation
Advice from social services	Advisory/conversation
Health seminars and education	Education
Nutrition advisory	Education
Patient education for back pain	Education
Diabetic training	Education
Education in sport and movement therapy	Education

### Ethical Considerations

A survey based on the developed questionnaire was reviewed for its ethical acceptability, particularly concerning the protection of participants’ social and psychological integrity by the Senate Commission for Research Ethics of Ostfalia University of Applied Sciences, University of Braunschweig/Wolfenbüttel. In accordance with national regulations and institutional policies, no institutional review board name or number was assigned, as the survey was anonymous and involved no interventions. The survey was conducted in accordance with the ethical principles of the Declaration of Helsinki. All participants provided informed consent prior to participation.

### Population

A total of 126 patients of a neurological rehabilitation clinic were interviewed with the questionnaire from 2013 to 2015. After data cleansing, 111 questionnaires could be used for analysis. In total, 15 questionnaires were refused or incomplete. Overall, 61 patients were evaluated with version 1.0 of the questionnaire, and 50 patients were evaluated with version 1.1 of the questionnaire. Patients’ diagnoses varied widely (stroke, intracerebral bleeding, encephalitis, myopathy, motoneuron disease, polyradiculitis, encephalomyelitis disseminata, myelopathy, and tumors of neurological tissue).

### Statistical Analysis

Nonparametric statistical methods were chosen due to the ordinal rating scale. Rank correlation with the Spearman ρ coefficient was used to assess the relationship between the subjective performance expectations for the 15 therapeutic fields, while the Mann-Whitney *U* test was used for group comparisons, and the chi-square test was used to compare the contingency of patients’ responses. Effect sizes were interpreted in accordance with Cohen [13]. All statistics were calculated with IBM SPSS Statistics (version 29). A critical level of *P*<.05 was considered significant for all statistics.

## Results

Patients’ demographic data are shown in Table 2. The patients’ age ranged from 32 to 86 (mean 65.3, SD 12.2) years. In total, 62 patients were male and 49 were female. The age distribution in the male and female groups was balanced (mean 65.4 vs 65.2, SD 11.5 y vs 13.1 years). The gender distribution in the age groups “<68 years” and “≥68 years” was similar, with slightly more males than females (male:female 31:26 versus 31:23).

**Table 2.** Demographic data.

	Total	Age (years)		Gender	
		<68	≥68	Male	Female
Age (years), mean (SD); range	65.3 (12.2); 32-86	55.8 (8.9); 32-67	75.4 (4.8); 68-86	65.4 (11.5); 32-86	65.2 (13.1); 33-85
Gender (male/female), n/n	62/49	31/26	31/23	—^a^	—

a Not applicable.

Overall, 56% of respondents were undergoing inpatient medical rehabilitation for the first time. Further, 38% of respondents had been to an inpatient medical rehabilitation facility at least twice, regardless of specialty or diagnosis, and 92% of respondents said they had not heard of virtual rehabilitation and needed an explanation. In these explanations, we followed the definition of VR in the context of neuroplasticity by Weiss et al [14]: “Virtual reality is defined as an approach to a user-computer interface that creates a real-time simulation of an environment, scenario, or activity, allowing the user to perform complex interactions using multiple sensory channels. Virtual rehabilitation training approaches use the latest VR technologies, improved robotic design, the development of haptic interfaces, and modern human-machine interactions for meaningful stimulation of the nervous system, thereby promoting brain plasticity.”

**Figure 1.**
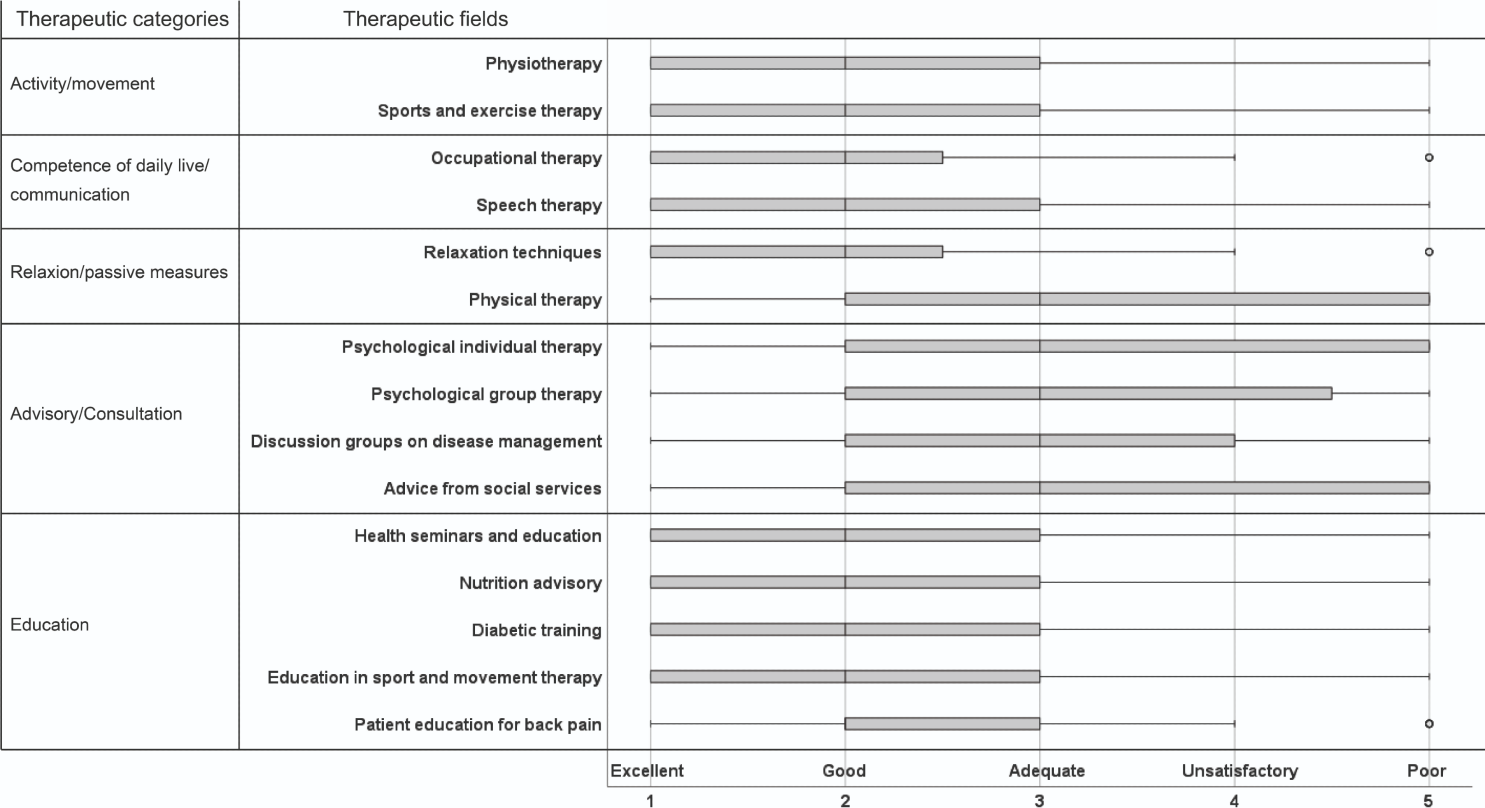
Distribution of patients’ evaluation of therapeutic fields with regard to possible benefits of using virtual reality.

An overview of the patients’ ratings is provided in Figure 1. In general, rehabilitants can imagine the use of VR in rehabilitation. This particularly applies to all items in the categories “activity/movement,” “competence in daily life/communication,” and “education” as well as for the occupational therapy (all these therapeutic measures have median score of 2, which represents the rating “good”), whereas physical therapy and all items in the category “advisory/conversation” exhibited a mean score of 3, which corresponds the rating “adequate.”

**Figure 2.**
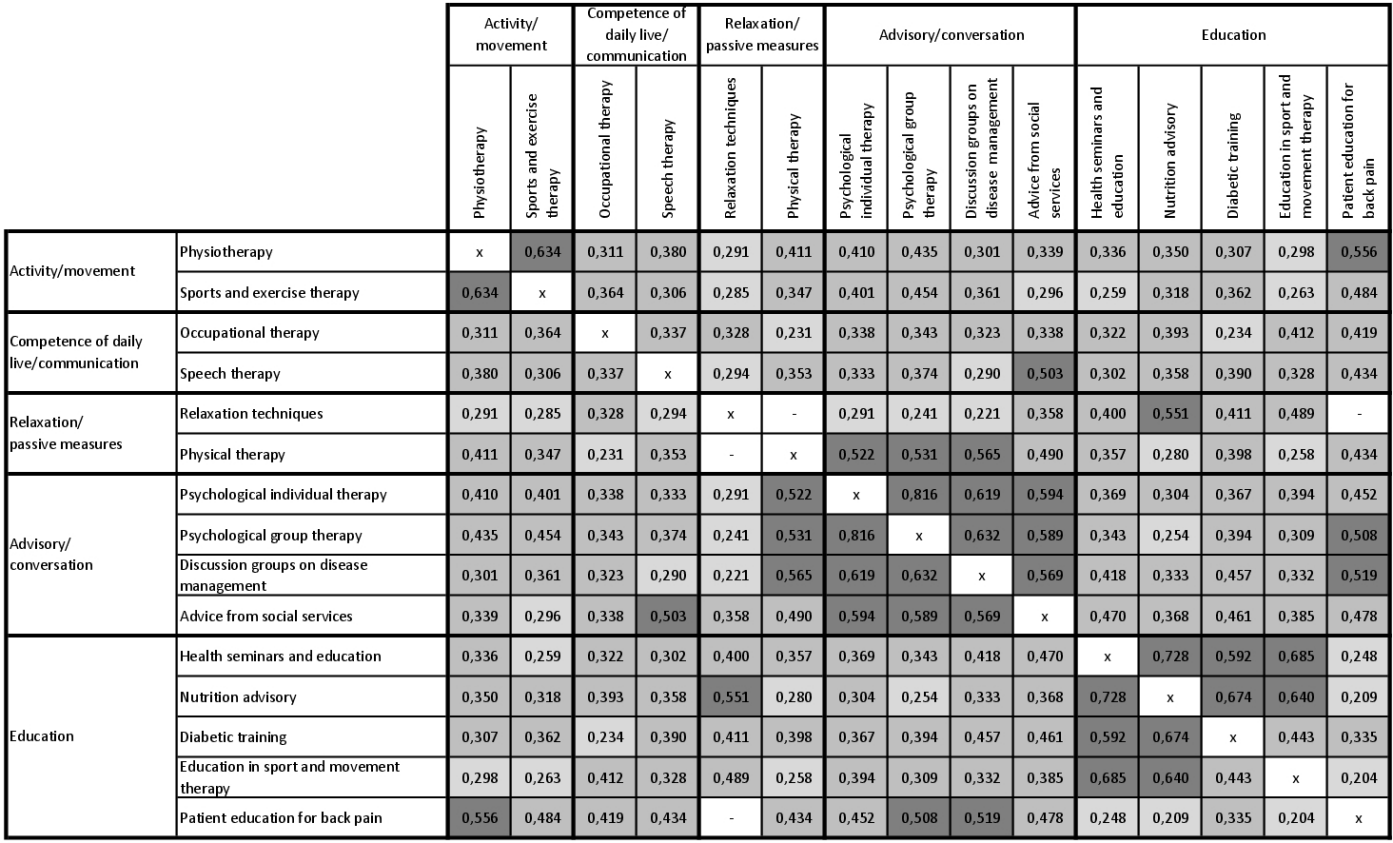
Rank correlation between patients’ evaluation of therapeutic fields and the monotonic relationship among the 15 therapeutic fields. Dark gray: large monotonic relationship (0.5≤|ρ|≤1.0); medium gray: medium monotonic relationship (0.3≤|ρ|<0.5); light gray: small monotonic relationship (small monotonic relationship); and white: no significant rank correlation.

The monotonic relationship among the 15 therapeutic fields is displayed in Figure 2. Except for the 2 combinations of therapeutic fields displayed in white, all correlations are significant at a .05 level (2-tailed). A rank correlation with a large effect size can be observed within the categories “activity/movement” and “advisory/conversation” as well within the category “education” but only among the therapeutic fields “health seminars and education,” “nutrition advisory,” and “diabetic training.” The monotonic relationship between the items of the category “competence in daily life/communication” is medium, while there is no significant rank correlation between “relaxation techniques” and “physical therapy.”

Table 3 shows the results of the group comparisons. There was no significant difference in the evaluation of VR between rehabilitants of employable age (aged <68 years) and those aged 68 years or older (Mann-Whitney *U* test, z=−.137 to −1.802, *P*=.07 to .90). Regarding the evaluation based on gender, it should be noted that male rehabilitation patients generally report greater subjective expectations for virtual rehabilitation than female patients, but there was only a significant difference with small effect sizes for “sports and exercise therapy,” “psychological individual therapy,” and “psychological group therapy.”

**Table 3.** Group comparisons (age and gender).

	Age			Gender		
	*P* value	z	Effect size r	*P* value	z	Effect size r
Physiotherapy	.21	−1267	—^a^	.07	1841	—
Sports and exercise therapy	.07	−1802	—	.006	−2726	.259
Occupational therapy	.82	−.222	—	.10	−1644	—
Speech therapy	.77	−.297	—	.43	−.786	—
Relaxation techniques	.20	−1291	—	.08	−1725	—
Physical therapy	.44	−.783	—	.28	−1092	—
Psychological individual therapy	.90	−.137	—	.007	−2699	.256
Psychological group therapy	.75	−.325	—	.005	−2761	.262
Discussion groups on disease management	.75	−.305	—	.21	−1256	—
Advice from social services	.50	−.677	—	.13	−1523	—
Health seminars and education	.13	−1506	—	.51	−.667	—
Nutrition advisory	.15	−1450	—	.38	−.871	—
Diabetic training	.71	−.378	—	.81	−.245	—
Education in sport and movement therapy	.13	−1501	—	.34	−.964	—
Patient education for back pain	.54	−.622	—	.20	−1288	—

a Not applicable.

Table 4 shows the distribution of patients’ answers regarding the additional questions. Overall, 78% of the patients believe, that VR can be used to achieve higher motivation and willingness to participate in medical rehabilitation therapy. There is no significant correlation with this answer and the experience with computers or game consoles (*P*=.08 [Fisher exact chi-square test]; expected cell frequencies were below 5).

**Table 4.** Patients’ responses to 2 questions: (1) Do you think you can achieve a higher motivation for cooperation and training using virtual reality? (2) Do you have any experiences with computers or game consoles? (Cross table; N=50).

	Do you have any experiences with computers or game consoles?, n (%)		
	Yes	No	Total
Do you think you can achieve a higher motivation for cooperation and training using virtual reality?			
Yes	27 (54)	12 (24)	39 (78)
No	4 (8)	7 (14)	11 (22)
Total	31 (62)	19 (38)	50 (100)

## Discussion

### Principal Findings

No other publication has addressed the subjective performance expectancy of virtual rehabilitation in rehabilitation patients. A possible limitation of this study is that the diagnoses of the rehabilitants and the amount of rehabilitation performed up to the time of the survey differed between the rehabilitants. The influence of this cofactors could not be evaluated with the given database. However, the results indicate a general willingness of rehabilitation patients to accept VR in the medical rehabilitation process. Since game consoles with motion-enhancing applications are well known in the population, it is not surprising that motion-enhancing VR is associated with a higher subjective performance expectancy than passive applications.

The results of the rank correlation suggest that when implementing VR strategies, certain areas of therapy could be linked for particular motivational support. This applies, for example, to activity/movement and education or competence in daily life/communication. A joint virtual therapeutic strategy for physical therapy and advisory/conversation is also conceivable.

Patients’ age and previous experience with computers or game consoles are not prerequisites for special motivation. This is an important aspect for the decision to treat certain patient groups separately. Measures with high information content and measures that require a high degree of imagination are also considered suitable for VR [15].

However, the responses represent only an imagined virtual rehabilitation. Patients were given a glimpse into the future. Ultimately, the concrete design of the motivating interaction, the social relationship, and the (immediate) reward determine the acceptance of new forms of therapy—virtual or real.

### Limitations

A major limitation of this study is that the survey was conducted 10 years ago. However, the authors still consider the prospective analysis of the existing data to be meaningful because no comparable studies have been published to date. Furthermore, existing health applications for virtual rehabilitation, such as the digital rehabilitation after care by CASPAR/MediClin GmbH [16], show, that the topic of subjective performance expectancy is highly relevant for current applications.

### Conclusion

Rehabilitation time is a valuable commodity, and rapid recovery means greater financial security for the individual and more lifetime in health. The benefits and efficiencies that VR can bring to various areas of medical rehabilitation need to be explored. Rehabilitation institutions are already gaining experience with professional systems or equipment from the gaming industry. However, considering the limited data available on acceptance, implementation and therapy outcomes have not yet been able to support large-scale industrial development and widespread use of virtual medical rehabilitation systems. To gain knowledge about the willingness of rehabilitation patients to accept VR systems, it was necessary to analyze their subjective performance expectations. This knowledge is also an important prerequisite for the acceptance of modern rehabilitation measures by health care payers and health insurance companies. An increasing trend toward the use of tele-rehabilitation confirms the results of the survey. The current trend toward virtual aftercare, especially via the internet, is showing increasing acceptance by both patients and health care payers.

## Abbreviations

VR: virtual reality
